# An Adaptation to Low Copper in *Candida albicans* Involving SOD Enzymes and the Alternative Oxidase

**DOI:** 10.1371/journal.pone.0168400

**Published:** 2016-12-29

**Authors:** Chynna N. Broxton, Valeria C. Culotta

**Affiliations:** Department of Biochemistry and Molecular Biology, Johns Hopkins University Bloomberg School of Public Health, Baltimore, MD, United States of America; Rutgers New Jersey Medical School, UNITED STATES

## Abstract

In eukaryotes, the Cu/Zn superoxide dismutase (SOD1) is a major cytosolic cuproprotein with a small fraction residing in the mitochondrial intermembrane space (IMS) to protect against respiratory superoxide. Curiously, the opportunistic human fungal pathogen *Candida albicans* is predicted to express two cytosolic SODs including Cu/Zn containing SOD1 and manganese containing SOD3. As part of a copper starvation response, *C*. *albicans* represses SOD1 and induces the non-copper alternative SOD3. While both SOD1 and SOD3 are predicted to exist in the same cytosolic compartment, their potential role in mitochondrial oxidative stress had yet to be investigated. We show here that under copper replete conditions, a fraction of the Cu/Zn containing SOD1 localizes to the mitochondrial IMS to guard against mitochondrial superoxide. However in copper starved cells, localization of the manganese containing SOD3 is restricted to the cytosol leaving the mitochondrial IMS devoid of SOD. We observe that during copper starvation, an alternative oxidase (AOX) form of respiration is induced that is not coupled to ATP synthesis but maintains mitochondrial superoxide at low levels even in the absence of IMS SOD. Surprisingly, the copper-dependent cytochrome c oxidase (COX) form of respiration remains high with copper starvation. We provide evidence that repression of SOD1 during copper limitation serves to spare copper for COX and maintain COX respiration. Overall, the complex copper starvation response of *C*. *albicans* involving SOD1, SOD3 and AOX minimizes mitochondrial oxidative damage whilst maximizing COX respiration essential for fungal pathogenesis.

## Introduction

Superoxide dismutases (SOD) play vital roles in the biology of reactive oxygen species (ROS) by disproportionating superoxide anion free radicals into hydrogen peroxide and molecular oxygen [1, 2]. There are three major SOD families including copper and zinc (Cu/Zn) SODs that use copper as the catalytic co-factor, a separate SOD family with either manganese or iron [3], and a rare family of nickel containing SODs [4]. Gram-negative bacteria restrict copper containing SODs to the periplasmic/extracellular space while manganese and iron SODs are intracellular/cytosolic [5, 6]. An analogous partitioning occurs in eukaryotic mitochondria where a Cu/Zn SOD (known as SOD1) lies in the intermembrane space (IMS) and a manganese SOD (known as SOD2) resides in the mitochondrial matrix [7–15]. SOD1 is also cytosolic in eukaryotes and its distribution between the cytosol and mitochondrial IMS involves a disulfide relay system and the copper chaperone for SOD1, CCS [16–18].

In mitochondria, superoxide anion is formed as a byproduct of respiration and is released into both the matrix and IMS where it reacts with SOD2 and SOD1 respectively [19, 20]. With conventional respiration involving the full electron transport chain and cytochrome c oxidase (COX), superoxide is released to the matrix by complex I [21], while complex III releases superoxide to both the IMS and matrix [19, 22]. COX is not the only form of respiration and certain fungi, plants, and protists express an alternative oxidase (AOX) that accepts electrons directly from coenzyme Q [23–29]. Unlike COX, AOX respiration is not coupled to ATP production [28]. One example of an organism that utilizes both COX and AOX respiration is the polymorphic fungus *Candida albicans* [30–33]. *C*. *albicans* is an opportunistic human pathogen that exists as a commensal of the human flora, but can become pathogenic in immune compromised individuals. Unlike fermenting yeasts such as *Saccharomyces cerevisiae*, *C*. *albicans* relies heavily on COX respiration for ATP [34, 35]. Thus, the rationale for retaining AOX respiration is not clear, particularly since AOX could potentially compete with COX for coenzyme Q electrons.

Aside from dual modes of respiration, *C*. *albicans* has an unusually large collection of six SOD enzymes including the Cu/Zn containing SOD1 [36, 37], the manganese containing SOD2 in the mitochondrial matrix [38], three extracellular copper-only SODs (SOD4,5,6) [39–42] and a second manganese containing SOD3 predicted to be cytosolic, as is the case with SOD1 [43]. We have recently shown that SOD1 and SOD3 are reciprocally expressed according to copper status: copper replete yeast only express SOD1 while copper starved cells repress SOD1 and induce SOD3 [44]. This switch in SOD enzymes is mediated by the copper sensing regulator MAC1 and is set into motion during fungal invasion of the kidney [44]. While both SOD1 and SOD3 are predicted to be cytosolic, it is unknown whether either can enter the mitochondrial IMS to deal with superoxide release in this compartment.

Herein we investigate the role of *C*. *albicans* SOD1 versus SOD3 in mitochondrial oxidative stress protection. We demonstrate that under copper replete conditions, the Cu/Zn containing SOD1 partitions between the cytosol and mitochondrial IMS and protects against mitochondrial superoxide. However, during copper starvation, the mitochondrial IMS becomes devoid of a SOD enzyme since the manganese containing SOD3 localizes exclusively to the cytosol. In spite of no IMS SOD, mitochondrial superoxide is not elevated. We find that during copper limitation, *C*. *albicans* induces AOX respiration, which suppresses mitochondrial superoxide and bypasses the need for an IMS SOD. In spite of extreme copper starvation, copper dependent COX respiration remains high and we provide evidence for a copper sparing mechanism whereby repression of SOD1 helps maintain high COX respiration in the face of copper starvation.

## Materials and Methods

### Yeast strains and culture conditions

*C*. *albicans* strains used in this study were derived from SC5314 including CA-IF100 (*arg4Δ/arg4Δ*, *leu2Δ/leu2Δ*::*cmLEU2*, *his1Δ/his1Δ*::*cdHIS1*, *URA3/ura3Δ*) and isogenic strains CA-IF001 (*sod1Δ*::*cmLEU2/sod1Δ*::*cdHIS1*) and CA-IF011 (*sod3Δ*::*cmLEU2/sod3 Δ*::*cdHIS1*) [41]. The SN152 strain (*his1Δ/his1Δ*, *leu2Δ/leu2Δ*, *arg4Δ/arg4Δ*, *URA3/ura3Δ*::*imm434*, *IRO1/iro1Δ*::*imm434*) and isogenic *mac1Δ*::*LEU2/mac1Δ*::*HIS1* strain were obtained from the Fungal Genetics Stock Center [45, 46]. The *ccs1Δ/Δ* strain *(ccs1Δ*::*cmLEU2/ccs1*::*cdHIS1)* was derived from SN78 (*ura3Δ*::*imm434/ura3Δ*::*imm434*, *leu2Δ/leu2Δ*, *his1Δ/his1Δ*) as previously described [47]. The strain constitutively expressing SOD1 was derived from CA-IF001 (*sod1Δ*::*cmLEU2/SOD1*) as previously described in [44]. In the so-called SOD1^con^ strain, the single allele of *SOD1* was engineered with a mutation in the MAC1 binding site at position +148 by gene replacement. A similar gene replacement was used to engineer the control SOD^rep^ with an intact MAC1 binding site [44].

*C*. *albicans* cells were cultured at 30°C in either enriched media (YPD; BD Difco) containing 1% yeast extract, 2% peptone, and 2% dextrose (w/v) or in a synthetic complete (SC) media with 0.67% yeast nitrogen base (US Biologicals) and 2% dextrose (w/v). Where indicated, cells were starved for copper by supplementing the growth media with 800 μM of the extracellular Cu(I) chelator BCS.

### Cell fractionation and analysis of SOD proteins and enzymatic activity

Mitochondria and post-mitochondrial supernatant (PMS)/largely cytosolic fractions were obtained from *C*. *albicans* essentially as described [31]. Briefly, cells were grown in YPD to an OD_600_ between 1.0 and 4.0, were harvested and successively washed in MilliQ deionized water and 0.1 M Tris-SO_4_ pH 9.4, 10 mM DTT, followed by incubation in the same buffer for 30 minutes at 30°C shaking at 100 RPM. Cells were harvested, and spheroplasts formed by incubating cells in 1.2 M sorbitol, 20 mM KH_2_PO_4_/K_2_HPO_4_ pH 7.4 with 0.3 mg/L Lyticase (Sigma). Spheroplasts were isolated by centrifugation at 1000 x g for 5 minutes and resuspended in 0.6 M sorbitol 20 mM K^+^HEPES pH 7.4 and lysed by dounce homogenization to create whole cell lysates. The lysate was spun at 12,000 x g for 10 minutes at 4°C to resolve PMS and crude mitochondria fractions. Mitochondria were washed twice in 1.2 M sorbitol 20 mM KH_2_PO_4_/K_2_HPO_4_ pH 7.4 buffer and where indicated, were resolved further into IMS and matrix fractions by resuspending in 20 mM K^+^ HEPES pH 7.4 and briefly vortexing for 5 seconds to rupture the outer membrane. The mitochondrial fraction was centrifuged at 12,000 x g for 10 minutes at 4°C to separate the matrix (pellet) from the soluble IMS. Prior to analysis by gel electrophoresis, crude whole mitochondria or matrix fractions were resuspended in a 10 mM sodium phosphate buffer 7.4 containing 0.1% Triton X-100 5 mM EDTA, 5 mM EGTA, 50 mM NaCl, 10% glycerol, a procedure that solubilizes the matrix and allows for visualization of matrix SOD2 [48].

SOD protein expression and localization was analyzed by immunoblot as previously described [44]. 70 μg of whole cell spheroplast lysates and the same cell equivalents of PMS and mitochondrial fractions were loaded onto 4–12% Bis-Tris gels (Thermo Fisher). Where indicated whole cell lysates of *C*. *albicans* were prepared by glass bead homogenization [44] and 30 μg of lysate protein was used. Blots were probed with anti-SOD1 and SOD3 primary antibodies as previously described [44]. Anti-SOD1 is a polyclonal antibody directed against *C*. *elegans* Sod-1 (JH766) that reacts well with copper and zinc containing SOD molecules across eukaryotes [48]. The anti-SOD3 polyclonal antibody is directed against a peptide unique to *C*. *albicans* SOD3 that is absent in mitochondrial SOD2 [47]. Anti-PGK1 at a 1:5000 dilution (Acris Antibodies, #AP21371AF-N) was used as a cytosolic marker. The polyclonal anti-SOD2 antibody is directed against *S*. *cerevisiae* SOD2 and was prepared as described [49]. This anti-SOD2 cross-reacts well with *C*. *albicans* SOD2 and was used at a 1:5000 dilution as a mitochondrial marker. On immunoblots, all four antibodies are highly specific. SOD enzymatic activity was analyzed by native gel electrophoresis and nitroblue tetrazolium staining as previously described [44] using 30 μg total cell lysate.

### RNA analysis

*C*. *albicans* cells were grown in SC medium in the absence or presence of 800 μM BCS to an OD_600_ between 1.0 and 2.0. RNA was isolated from 20 OD_600_ cell units using an RNeasy mini kit (Qiagen), and then subsequently converted to cDNA using Superscript IV Reverse Transcriptase kit (Thermo Fisher Scientific). Real time PCR was performed as previously described [44]. cDNA was diluted 20-fold before PCR amplification with iQ SYBR Green Supermix (Bio-Rad) and values were normalized to *TUB2* transcripts in each sample. The *SOD3* and *TUB2* primers were used as described [44]; *AOX2* primers are GTTGGTCAAGGGGTTTTCACTAATG and ACTGCCACTTCAGGGATTTTCATGG.

### Measurements of mitochondrial superoxide, oxygen consumption and cellular copper

Mitochondrial superoxide was measured in *C*. *albicans* cells initially cultured in YPD media to early stationary phase (OD_600_ 6.0–10.0), then diluted back to OD_600_ ≈0.1 in SC medium and grown to OD_600_ of 1.0–2.0. Background fluorescence is minimized in cells from SC medium. 5 μM of MitoSOX Red (Life Technologies) in DMSO or the equivalent volume of DMSO control was added to 500 μl of culture and incubated for 45 minutes in round bottom tubes at 250 rpm in the dark at 30°C. Where indicated, cells were co-incubated with MitoSOX red and 500 nM MitoTracker Green FM (Thermo Scientific). To monitor effects of AOX inhibition, cultures were incubated for 1 hour with 5 mM SHAM prior to MitoSOX addition. Cells were washed three times in phosphate buffered saline (PBS), resuspended in PBS and subjected to either microscopic visualization of MitoSOX Red fluorescence using a Zeiss Observer Z1 fluorescence microscope with a Zeiss Plan-Apochromat 63x objective, or to fluorescence quantification on a Biotek Synergy HT microplate reader with excitation and emission at 510 nm and 580 nm respectively. Fluorescence quantification used 2 x 10^7^ of cells in 200 μl PBS.

Oxygen consumption measurements were conducted on whole cells essentially as described [35] using cells grown in YPD to early log (OD_600_ of 1.0–2.0). Ten OD_600_ units of cells were harvested, washed once in PBS and resuspended in 2 ml of YPD. Oxygen consumption was measured polarographically using a Clark-type oxygen electrode in a magnetically stirred, thermostatically controlled 1.5-ml chamber at 25°C (Oxytherm; Hansatech). Following 1 min, 5mM salicylhydroxamic acid (SHAM) was added to the oxygen chamber to inhibit AOX respiration followed by addition of 1mM potassium cyanide (KCN) to inhibit COX respiration. The change in oxygen saturation over time was used to calculate rates of oxygen consumption. The degree to which the oxygen consumption rate is inhibited by SHAM or KCN was used to derive the percent oxygen consumption by AOX or COX, respectively.

Copper content of whole cells was measured on the same cultures used for oxygen consumption. 20 OD_600_ units of cells were washed twice in 10 mM Tris pH 8.0 1 mM EDTA, and twice in deionized water. Cells were resuspended in 500 μl MilliQ deionized water before analysis of copper by atomic absorption spectroscopy (AAS) on a PerkinElmer Life Sciences AAnalyst 600 graphite furnace instrument as described [44].

## Results

### A fraction of *Candida albicans* SOD1, but not SOD3, localizes to the mitochondrial intermembrane space.

**Fig 1:**
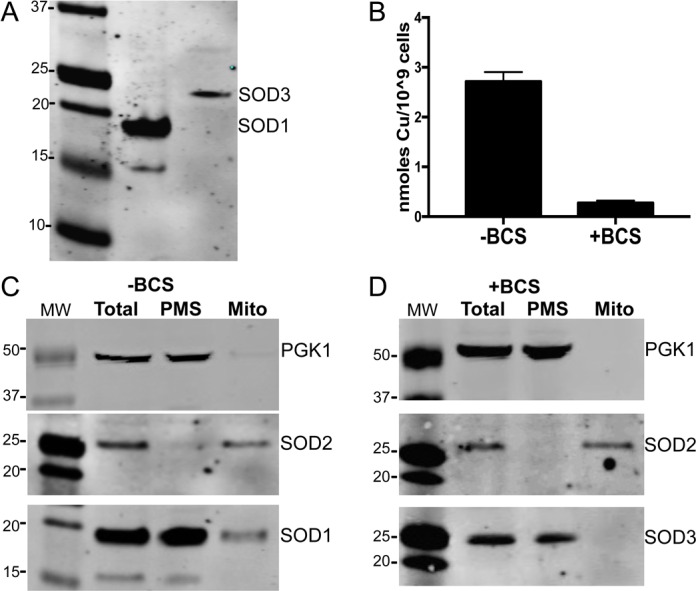
A fraction of *C*. *albicans* SOD1 but not SOD3 localizes to the mitochondria IMS. *C*. *albicans* strain CAIF-100 was grown in enriched medium in either the presence or absence of 800 μM of the extracellular Cu(I) chelator BCS as indicated. (A) Lysates were prepared from spheroplasts and were analyzed for SOD1 and SOD3 by immunoblot. (B) Total cellular copper was measured by atomic absorption spectroscopy. (C-D) Spheroplast cell lysates (Total) were fractionated into the largely cytosolic post-mitochondrial supernatant (PMS) and crude mitochondria (mito) by differential centrifugation. 70 μg of total lysate protein and the same cell equivalents of PMS and mitochondria were analyzed by immunoblot for SOD2 (marker of mitochondrial matrix), PGK1 (marker of cytosol) and either SOD1 (C) or SOD3 (D). MW = molecular weight markers. SOD1 often appears as two bands, both of which are down-regulated by copper starvation.

In standard enriched medium, *C*. *albicans* cells are copper replete and only express SOD1, not SOD3 (Fig 1A). Supplementing cultures with the extracellular Cu(I) chelator BCS (bathocuproinedisulfonic acid) results in a ten-fold reduction in intracellular copper (Fig 1B), and cells respond to this copper starvation stress by repressing SOD1 and inducing the manganese containing SOD3 (Fig 1A) [44]. Using these differential copper conditions we examined the localization of SOD1 versus SOD3.

**Fig 2:**
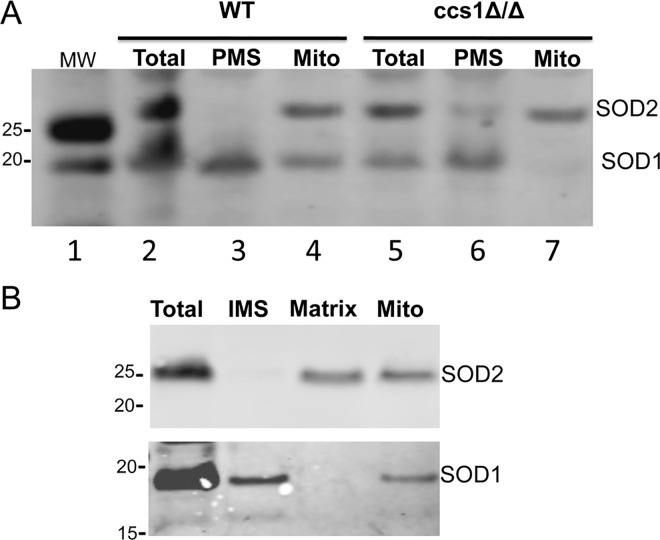
*C*. *albicans* SOD1 localizes to the mitochondrial IMS and requires its copper chaperone CCS1 for mitochondrial localization. (A) Cell lysates from wild type (CAIF-100) and the isogenic *ccs1Δ/Δ* strain were fractionated into PMS and mitochondria and analyzed by immunoblot for SOD1 and SOD2. (B) The mitochondrial fraction derived from 70 μg total lysate protein was further fractionated into IMS and matrix as described in *Materials and Methods* and analyzed for SOD1 and SOD2 by immunoblot. Numbers on left indicate molecular weight markers.

We first addressed whether the Cu/Zn containing SOD1 partitions between the cytosol and mitochondria as was shown for *S*. *cerevisiae* and mammalian cells [8, 9, 50]. Whole cell lysates were subjected to differential centrifugation to resolve crude mitochondria from the largely cytosolic or post mitochondrial supernatant (PMS) fraction. Identical cell equivalents of each fraction were analyzed by western blot. As seen in Fig 1C, the majority of SOD1 fractionates with the cytosolic maker PGK1, consistent with a largely cytosolic localization of the protein. We also observed a small fraction of SOD1 partitioning with the mitochondria together with the mitochondrial marker, SOD2. By quantification of the results from five experimental trials, we find that mitochondrial SOD1 represents 5% of total cellular SOD1 (standard deviation = 0.0247). This value is quite comparable to the ≈1–5% reported for human and *S*. *cerevisiae* mitochondrial SOD1 [8, 14]. As was shown for human and *S*. *cerevisiae* SOD1 [16, 51], *C*. *albicans* SOD1 appears to require its copper chaperone CCS for mitochondrial uptake, as SOD1 was not detected in mitochondria from *ccs1Δ/Δ* cell (Fig 2A, compare lanes 4 and 7). Additionally, upon fractionation of IMS and matrix components, we observed that SOD1 localizes to the IMS while the manganese containing SOD2 partitions to the mitochondrial matrix (Fig 2B), precisely as was shown for *S*. *cerevisiae* and mammalian SODs [8–11, 13–15]. Altogether, the studies of Figs 1C and 2 demonstrate that under copper replete conditions, *C*. *albicans* parallels other eukaryotes by importing a small fraction of SOD1 into the mitochondrial IMS.

We next addressed the status of *C*. *albicans* mitochondria when cells are starved for copper and switch from Cu/Zn containing SOD1 to the manganese containing SOD3. In the experiment of Fig 1D, the mitochondrial and PMS fractions of copper starved cells were probed for SOD3. We observed that unlike SOD1, SOD3 was only visible in the PMS, and over three experimental trials there was no detectable SOD3 in the mitochondrial fraction (representative result shown in Fig 1D).

### Mitochondrial superoxide under copper replete versus copper starvation conditions

**Fig 3:**
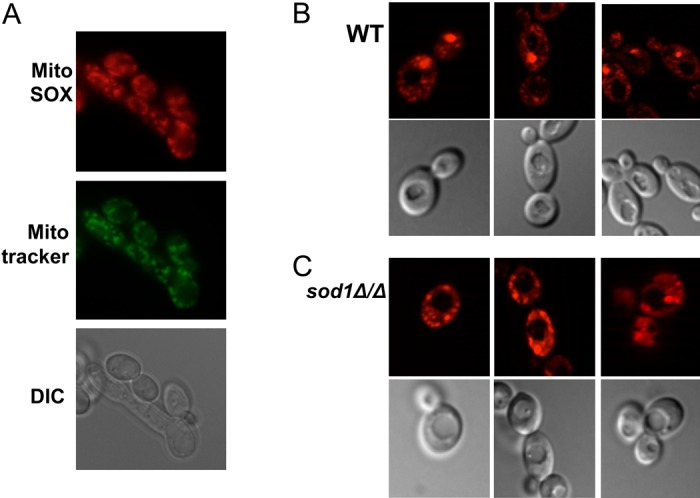
MitoSOX Red as a probe for mitochondrial superoxide in *C*. *albicans*. Log phase CA-1F100 *C*. *albicans* wild type cells (A, B) or the isogenic *sod1Δ/Δ* strain (C) grown under non-stress conditions were incubated with 5 mM MitoSOX Red as a marker for mitochondrial superoxide and imaged by fluorescence microscopy at 63X. (A) “DIC” = light microscopy images and “MitoTracker” = cells treated with both MitoSox Red and 500 μM MitoTracker Green as a marker for mitochondria. (B,C) Wild type and *sod1Δ/Δ* cells were examined in parallel where the top and bottom rows show representative MitoSox Red fluorescence and DIC images, respectively.

**Fig 4:**
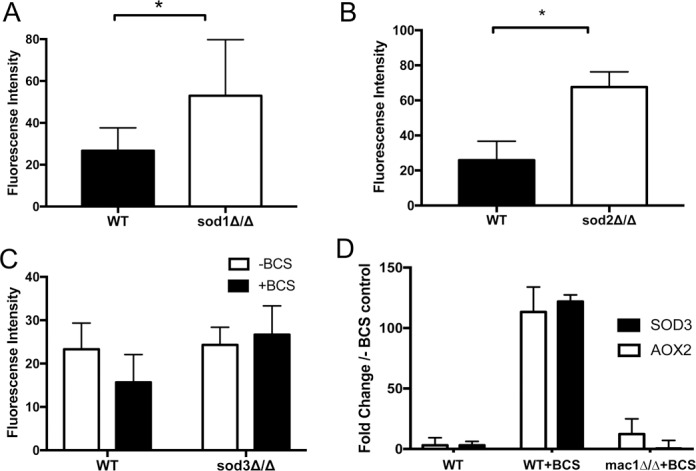
Effects of copper starvation and *sod* gene mutations on mitochondrial superoxide and *AOX2* expression. (A-C) MitoSOX Red fluorescence was measured spectrophotometrically at 580 nm where fluorescence intensity is the signal obtained with 2x10^7^ cells as described in *Materials and Methods*. (A-B) A comparison of the effects of *sod1Δ/Δ* versus *sod2Δ/Δ* mutations on MitoSOX Red fluorescence where results represent the averages of eight and four independent samples respectively, and statistical significance was determined using a paired two-tailed Student’s t-test. Asterisks indicate statistical significance with P values < 0.05. Error bars represent standard deviation. The baseline MitoSOX Red fluorescence of non-stressed wild type cells typically varies between 20–40 fluorescent units, but regardless of this baseline, *sod1Δ/Δ* and *sod2Δ/Δ* mutants exhibited enhanced fluorescence. (C) The indicated strains were grown in the presence or absence of 800 μM BCS as designated. Results represent the averages of three independent cultures where error bars are standard deviation. The difference between minus and plus BCS with the wild type strain is not statistically significant (p = 0.2063). (D) *AOX2* and *SOD3* mRNA were quantified by qRT-PCR from wild type (SN152) and the isogenic *mac1Δ/Δ* strain as described in *Materials and Methods*. Values are normalized to that obtained with wild type SN152 *C*. *albicans* cells without BCS. Shown are averages of two biological replicates where error bars are standard deviation.

We sought to understand the consequences of no IMS SOD during copper starvation. To monitor mitochondrial superoxide we utilized the superoxide specific fluorescent probe MitoSOX Red, a derivative of dihydroethidium designed to target mitochondria. As seen in Fig 3A, MitoSOX yields a punctate pattern of fluorescence in *C*. *albicans* wild type cells mirroring that of MitoTracker Green FM, validating its use as a specific marker of mitochondrial ROS in *C*. *albicans*. Under copper replete conditions, *sod1Δ/Δ* mutants also display a punctate pattern of fluorescence (Fig 3C) that appeared enhanced compared to the signal obtained with wild type cells analyzed in parallel (Fig 3B). To quantitate any changes between wild type and *sod1Δ/Δ* cells, MitoSOX fluorescence was measured spectrophotometrically at 580 nm. Over four experimental trials with eight independent samples, *sod1Δ/Δ* cells consistently exhibited a ≈2- fold increase in MitoSOX detectable fluorescence (Fig 4A). The MitoSOX signal was also increased in *sod2Δ/Δ* cells lacking the mitochondrial matrix SOD2 (Fig 4B). Thus, MitoSOX Red is an effective indicator of mitochondrial ROS in *C*. *albicans*, sensitive to both IMS (SOD1-relevant) and mitochondrial matrix (SOD2-relevant) superoxide.

Using MitoSOX Red, we probed the mitochondrial ROS status of copper deficient cells. We anticipated an elevation in mitochondrial superoxide, since the mitochondrial IMS appears devoid of a SOD enzyme under these conditions. However, MitoSOX Red fluorescence was not elevated in either wild type cells or *sod3Δ/Δ* strains treated with BCS (Fig 4C) consistent with the notion that SOD3 does not enter the mitochondria. It was therefore possible that copper starvation stress induces a non-SOD antioxidant to minimize mitochondrial ROS.

### Modes of mitochondrial respiration under copper replete versus copper starvation conditions

As mentioned above, *C*. *albicans* uses two forms of respiration: COX respiration that employs the full electron transport chain and is a major source of cellular ATP, and AOX respiration that accepts electrons directly from coenzyme Q and is not coupled to ATP production [23, 30, 31]. *C*. *albicans* expresses dual AOX isoforms, *AOX1* and *AOX2* [32, 33], and *AOX2* was previously shown to be a target of the same MAC1 regulator that controls *SOD1* and *SOD3* expression [52]. Copper regulation of *AOX2* had not been previously reported and we find using quantitative real-time PCR a very strong induction of *AOX2* mRNA in cells starved for copper (Fig 4D). This pronounced induction of *AOX2* mimics that of *SOD3* mRNA and is abolished by *mac1Δ/Δ* mutations (Fig 4D).

**Fig 5:**
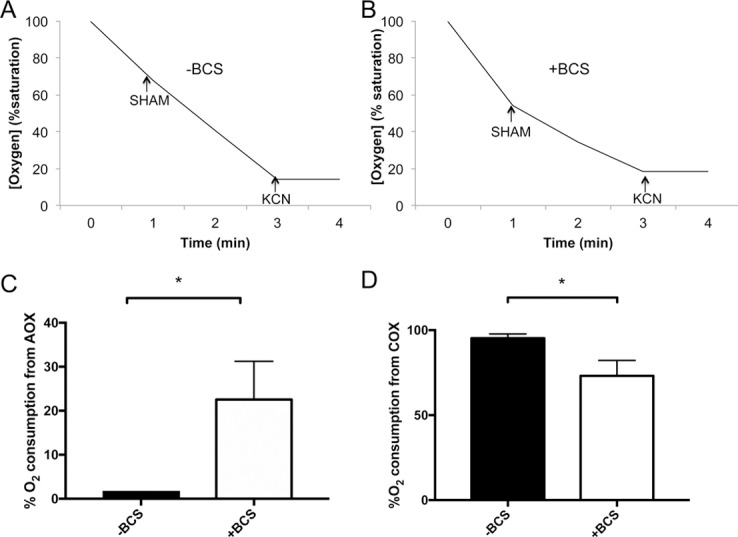
AOX respiration contributes to oxygen consumption during copper starvation. *C*. *albicans* strain CA-IF100 grown in the presence or absence of BCS was subjected to measurements of oxygen consumption using a Clark electrode. (A,B) Shown are representative profiles of oxygen consumption as monitored by percent oxygen saturation in solution. At the indicated time points, cells were treated with 5 mM SHAM to inhibit AOX respiration followed by 1 mM KCN to inhibit COX respiration. (C,D) The rates of oxygen consumption were derived from oxygen saturation curves. Plotted are the percentages of oxygen consumption inhibited by SHAM and attributed to AOX (C) and the oxygen consumption inhibited by KCN and attributed to COX (D). Results represent the averages of three independent samples with error bars representing standard deviation. Statistical significance was determined using a paired two-tailed Student’s t-test. Asterisks indicate statistical significance with P values < 0.05.

We tested whether the induction of *AOX2* mRNA correlates with utilization of the AOX pathway for respiration. Fig 5A and 5B shows representative oxygen consumption experiments where cells were successively treated with salicylhydroxamic acid (SHAM) to inhibit AOX respiration followed by treatment with potassium cyanide KCN to inhibit COX [31]. By calculating the rates of oxygen consumption in the presence and absence of these inhibitors, the fraction of respiration due to AOX versus COX can be ascertained. We find that during copper replete conditions, COX respiration accounts for essentially all of the oxygen consumption while AOX respiration is undetectable (Fig 5C and 5D). However, during copper starvation, there was a pronounced induction of AOX respiration, accounting for nearly 25% of total oxygen consumption; COX respiration proportionately declined (Fig 5C and 5D). AOX respiration is clearly induced during copper starvation.

**Fig 6:**
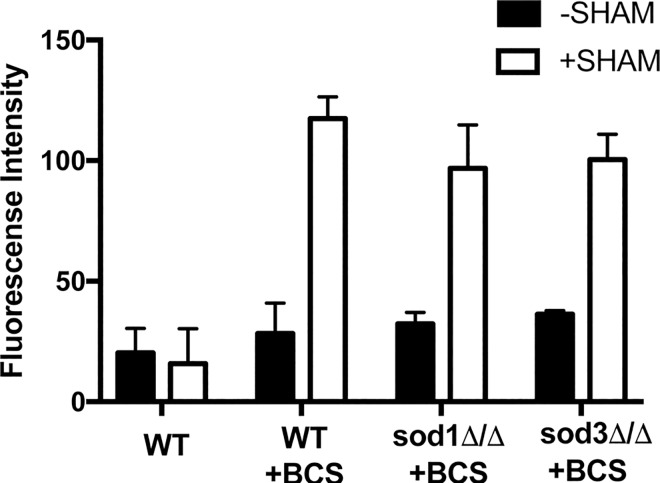
Effect of inhibiting AOX respiration on mitochondrial superoxide under copper starvation. MitoSOX Red fluorescence was measured in the designated strains. Where indicated, cells were treated with 5 mM SHAM for 1 hour to inhibit AOX respiration prior to MitoSOX Red incubation. Results represent the averages of two independent cultures with error bars representing standard deviation.

AOX respiration bypasses complex III of the electron transport chain, and since complex III is a source of IMS superoxide [19], AOX induction could conceivably minimize mitochondrial ROS. To test the impact of AOX on mitochondrial superoxide, MitoSOX Red fluorescence was measured in cells in which AOX was inhibited by SHAM. As seen in Fig 6, SHAM had no impact on mitochondrial ROS in copper replete wild type cells, consistent with the observation of no AOX respiration under these conditions (Fig 5B). However, SHAM treatment had a marked effect on mitochondrial ROS under copper starvation conditions. In wild type cells, MitoSOX Red fluorescence was enhanced 3–4 fold when AOX was inhibited with SHAM (Fig 6). The same is true of *sod1Δ/Δ* and *sod3Δ/Δ* strains starved for copper, demonstrating that the change in superoxide occurs independent of these SOD enzymes (Fig 6). Together, the studies of Figs 5 and 6 demonstrate that during copper starvation, *C*. *albicans* induces AOX respiration to reduce mitochondrial superoxide.

### Evidence for copper sparing with SOD1

Since cytochrome c oxidase is a copper dependent enzyme, it was curious that COX respiration falls no more than 30% (Fig 5D) when intracellular copper levels drop by ≥90% (Fig 1B). Under these same copper starvation conditions, activity of copper dependent SOD1 is undetectable, largely due to *SOD1* gene repression. Does this repression of *SOD1* serve to spare copper for COX? To address this, we used a *C*. *albicans* strain engineered to constitutively express *SOD1*.

**Fig 7:**
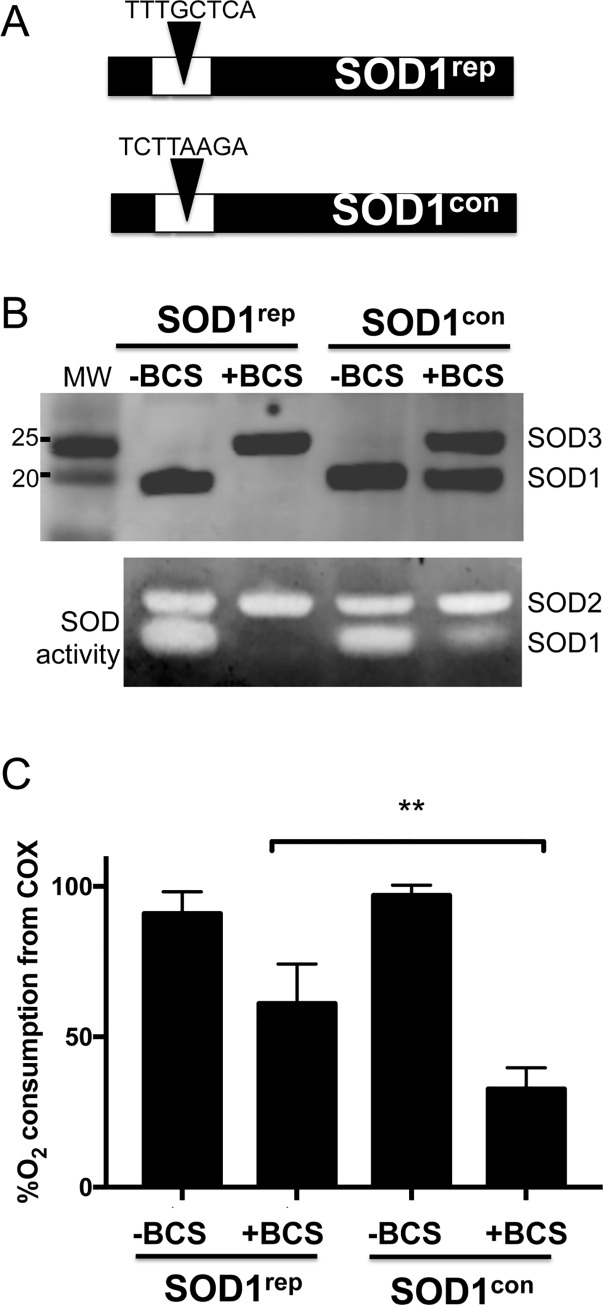
Constitutive expression of SOD1 inhibits COX repression during copper starvation. (A) Schematic showing the coding region (black) and intron (white) of the *SOD1* gene with sequence of the wild type and mutated MAC1 sites in SOD1^rep^ (*SOD1* repressed by copper) and SOD1^con^ (*SOD1* constitutively expressed). (B) Strains expressing a single copy of either SOD1^rep^ or SOD1^con^ were grown in the presence or absence of BCS and whole cell lysates were (top) immunoblotted for SOD1 and SOD3 as in Fig 1A and (bottom) subjected to SOD enzymatic activity analysis by the native gel assay. Numbers on left of immunoblot represent molecular weight markers and the positions of SOD1 and SOD2 migration on the native gel are indicated on the right. Densitometric tracings show that the intensity of SOD1 activity in SOD1^con^ +BCS is 38% that of the corresponding–BCS sample. (C) Cytochrome c oxidase respiration was measured as in Fig 5D as a function of KCN inhibitable oxygen consumption. Results represent the averages of two independent experiments with a total of four biological replicates with error bars representing standard deviation. Statistical significance was determined using a paired two-tailed Student’s t-test. Asterisks indicate statistical significance with P values < 0.005.

*C*. *albicans SOD1* contains a single intronic MAC1 site that mediates *SOD1* repression during copper starvation [44] (illustrated in Fig 7A). As seen in Fig 7B top, mutating this MAC1 site in chromosomal *SOD1* results in constitutive *SOD1* expression and cells express both SOD1 and SOD3 with copper starvation, consistent with previous findings [44]. We observe that constitutively expressed SOD1 is not fully active with copper starvation due to limitation of its copper co-factor; nevertheless it can secure sufficient copper for 30–40% of the activity seen with copper replete conditions (Fig 7B bottom and legend). We tested whether this limited copper activation of SOD1 can impact COX respiration. As seen in Fig 7C, the control strain expressing repressible *SOD1* with intact MAC1 site exhibited the anticipated ≈30% decrease in COX respiration with copper starvation. By comparison, COX respiration drops ≈60% with copper starvation in the strain that constitutively expresses SOD1. Thus, even a small retention in SOD1 activity during copper starvation conditions is sufficient to inhibit COX respiration. We conclude that the repression of SOD1 during copper starvation helps maximize COX respiration.

## Discussion

**Fig 8:**
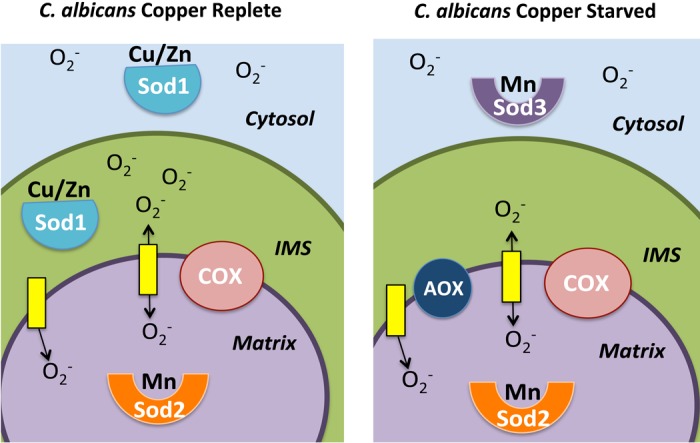
Adaption to copper in *Candida albicans* involving SOD enzymes and the mode of mitochondrial respiration. Cartoon to illustrate *C*. *albicans* adaptation to low copper by alternating SOD enzymes and inducing AOX respiration. When copper is abundant (left), *C*. *albicans* utilizes COX respiration and SOD1 is active in both the IMS and the cytosol. During copper starvation (right), cells switch to expressing SOD3 in the cytosol, but the IMS becomes devoid of a SOD enzyme. AOX respiration is induced which helps to lower IMS superoxide. Regardless of copper conditions, COX respiration remains active and mitochondrial matrix superoxide is managed by SOD2.

Copper is an essential nutrient of restricted availability and when cellular copper declines, priorities must be established to spare the nutrient. In the case of *C*. *albicans*, the response to low copper involves down-regulating the cuproprotein SOD1. Like other eukaryotes, SOD1 resides in both the *C*. *albicans* cytosol and mitochondrial IMS, and when copper declines, *C*. *albicans* maintains ROS homeostasis by replacing SOD1 with two copper-independent enzymes including SOD3 in the cytosol and AOX in the mitochondria (see model, Fig 8). We show here that AOX serves to minimize mitochondrial ROS in the absence of an IMS SOD enzyme. The role of SOD3 in the cytosol is less clear, but may act to substitute for SOD1 in cell signaling. Previously, we have shown that in *S*. *cerevisiae*, cytosolic SOD1 functions in glucose sensing and signaling [53], and the laboratories of Thiele and Zheng have shown a role for nuclear SOD1 in gene regulation and the response to DNA damage [54, 55]. *C*. *albicans* SOD3 could very well replace SOD1 in one or more of these extra-mitochondrial roles in signaling through ROS.

We propose that the down-regulation of *C*. *albicans* SOD1 during copper starvation serves to free up substantial metal for other processes. We find that copper-dependent COX respiration remains high in *C*. *albicans* during copper starvation; however this activity can be compromised when even a small level of copper is distributed to SOD1, as was found using a constitutively expressed allele of *SOD1*. By down-regulating SOD1 during copper starvation, *C*. *albicans* can spare this metal for COX respiration, crucial for this opportunistic fungal pathogen that relies heavily on COX respiration for ATP. The notion of sparing copper for respiration is reminiscent of what has been reported by Merchant and colleagues for the photosynthetic algae, *Chlamydomonas reinharti* [56]. *C*. *reinhardtii* does not express a copper requiring SOD1 [57], rather a major source of cellular copper is plastocyanin used for photosynthesis. In this organism when copper levels are low, plastocyanin is degraded and allows for copper to be allocated for the synthesis of COX [56]. To compensate for loss of plastocyanin, *C*. *reinhardtii* induces iron requiring Cytochrome c_6_ [58, 59], analogous to the induction of the non-copper alternatives SOD3 and AOX that substitute for SOD1 in *C*. *albicans*.

AOX respiration is not coupled to ATP synthesis, and the rationale for retaining this apparently futile mode of oxygen consumption has been the subject of much investigation. Many previous studies support a role for AOX in offsetting certain defects associated with inhibition of COX respiration. By directly accepting electrons from coenzyme Q, AOX can help maintain the NAD/NADH balance through complex I activity [60] or prevent superoxide formation when downstream portions of the electron transport chain are interrupted [61–63]. Consistent with its role in correcting COX deficiencies, AOX gene expression is induced by inhibitors of respiration in several organisms [64–66]. Here we provide a new layer to AOX functionality, specifically under copper limiting conditions. In *C*. *albicans*, AOX not only offsets deficiencies in COX respiration but compensates for loss of IMS SOD1 by minimizing mitochondrial ROS. Inside the animal host, *C*. *albicans* is subject to great fluctuations in copper availability and experiences copper starvation stress during invasion of the kidney [44]. The adaptation described here involving SOD1, SOD3, AOX and COX is expected to minimize mitochondrial oxidative damage while maximizing COX respiration required for pathogenesis [34, 35].

Lastly, why has *C*. *albicans* retained the copper containing SOD1 when the fungus is susceptible to copper starvation stress *in vivo* and the requirement for SOD1 appears to be bypassed by cytosolic Mn SOD3 and mitochondrial AOX? Our previous work in *S*. *cerevisiae* has shown that in addition to scavenging superoxide, SOD1 serves as a copper buffer and can minimize copper toxicity in cases of copper excess [67]. *C*. *albicans* SOD1 may have a similar role in copper detoxification. In the case of *Chlamydomonas*, it has been suggested that Cu-plastocyanin acts as a storage depot for copper that can be rapidly allocated to COX when copper becomes limiting [56]. By the same token, SOD1 may temporarily hold copper that is re-distributed to COX or other sites as needed.
